# Metagenomics Reveals Diet-Specific Specialization of Bacterial Communities in Fungus Gardens of Grass- and Dicot-Cutter Ants

**DOI:** 10.3389/fmicb.2020.570770

**Published:** 2020-09-24

**Authors:** Lily Khadempour, Huan Fan, Ken Keefover-Ring, Camila Carlos-Shanley, Nilson S. Nagamoto, Miranda A. Dam, Monica T. Pupo, Cameron R. Currie

**Affiliations:** ^1^Department of Bacteriology, University of Wisconsin-Madison, Madison, WI, United States; ^2^Department of Energy Great Lakes Bioenergy Research Center, University of Wisconsin-Madison, Madison, WI, United States; ^3^Department of Earth and Environmental Sciences, Rutgers University, Newark, Newark, NJ, United States; ^4^Center for Integrative Conservation, Xishuangbanna Tropical Botanical Garden, Chinese Academy of Sciences, Yunnan, China; ^5^Departments of Botany and Geography, University of Wisconsin-Madison, Madison, WI, United States; ^6^Department of Biology, Texas State University, San Marcos, TX, United States; ^7^Department of Plant Protection, São Paulo State University, Botucatu, Brazil; ^8^Department of Nutritional Sciences, University of Wisconsin-Madison, Madison, WI, United States; ^9^School of Pharmaceutical Sciences of Ribeirão Preto, University of São Paulo, Ribeirão Preto, Brazil

**Keywords:** *Atta*, herbivory, microbiome, substrate specialization, KEGG, attine ant

## Abstract

Leaf-cutter ants in the genus *Atta* are dominant herbivores in the Neotropics. While most species of *Atta* cut dicots to incorporate into their fungus gardens, some species specialize on grasses. Here we examine the bacterial community associated with the fungus gardens of grass- and dicot-cutter ants to examine how changes in substrate input affect the bacterial community. We sequenced the metagenomes of 12 *Atta* fungus gardens, across four species of ants, with a total of 5.316 Gbp of sequence data. We show significant differences in the fungus garden bacterial community composition between dicot- and grass-cutter ants, with grass-cutter ants having lower diversity. Reflecting this difference in community composition, the bacterial functional profiles between the fungus gardens are significantly different. Specifically, grass-cutter ant fungus garden metagenomes are particularly enriched for genes responsible for amino acid, siderophore, and terpenoid biosynthesis while dicot-cutter ant fungus gardens metagenomes are enriched in genes involved in membrane transport. Differences between community composition and functional capacity of the bacteria in the two types of fungus gardens reflect differences in the substrates that the ants incorporated. These results show that different substrate inputs matter for fungus garden bacteria and shed light on the potential role of bacteria in mediating the ants’ transition to the use of a novel substrate.

## Introduction

Understanding the role of microbial symbionts in aiding nutrient acquisition is fundamental to understanding the biology of herbivores. Most herbivores host microbial symbionts that serve as an interface between them and the plants that they consume. These microbes can compensate for the hosts’ lack of physiological capacity to obtain energy and nutrients from plants (Hansen and Moran, 2013). Herbivore microbial symbionts, often residing in the guts of animals, have been implicated in aiding plant biomass breakdown (Talbot, 1977; Kudo, 2009; Adams et al., 2011; Hess et al., 2011), plant defense compound remediation (Wang et al., 2012; Adams et al., 2013; Boone et al., 2013), and nutrient supplementation (Warnecke et al., 2007; Hansen and Moran, 2011; LeBlanc et al., 2013). Microbial communities differ between hosts that specialize on different substrates (Muegge et al., 2011), and changes in these communities and their functional capacity are integral to their hosts’ transition to utilizing novel substrates (Delsuc et al., 2013; Kohl et al., 2014; Hammer and Bowers, 2015; Li et al., 2015; Kohl and Yahn, 2016).

Leaf-cutter ants represent a paradigmatic example of the microbial mediation of herbivory. They are dominant herbivores in the Neotropics, consuming up to an estimated 17% of foliar biomass in the systems in which they live (Herz et al., 2007; Costa et al., 2008). These ants have significant impact on their surrounding ecosystems, due to the volume of plant biomass they consume and soil that they excavate in building their underground colonies (Fowler et al., 1986; Moutinho et al., 2003; Gutiérrez and Jones, 2006; Herz et al., 2007; Costa et al., 2008). Leaf-cutter ants lack the capacity to break down recalcitrant plant material. Instead, they farm a fungus, *Leucoagaricus gongylophorus*, which enzymatically breaks down recalcitrant biomass in the leaf material that the ants forage (Kooij et al., 2011; Nagamoto et al., 2011; Suen et al., 2011a; Aylward et al., 2013; Grell et al., 2013; Khadempour et al., 2016). *Leucoagaricus gongylophorus* produces gongylidia, specialized hyphal swellings that contain an abundance of sugars and lipids, that the ants consume and feed to larvae (Bass and Cherrett, 1995; North et al., 1997). In the leaf-cutter ant system, the fungus garden serves as the ants’ external gut (Aylward et al., 2012b; Khadempour et al., 2016).

Previous studies revealed that a community of bacteria reside within leaf-cutter ant fungus gardens (Scott et al., 2010; Suen et al., 2010; Aylward et al., 2012a; Meirelles et al., 2016; Moreira-Soto et al., 2017). These communities are dominated by Gammaproteobacteria, and consistently contain strains of *Pseudomonas*, *Enterobacter* and either *Rahnella* or *Pantoea*, and are highly similar to communities of bacteria associated with other fungus-farming insects (Suen et al., 2010; Aylward et al., 2012a, 2014). Some garden bacteria are vertically transmitted, maternally through the fungus pellets that alate queens use to establish new fungus gardens (Moreira-Soto et al., 2017). The consistency and vertical transmission of the bacterial communities, suggest that they are important to the fitness of their hosts. One study, by Pinto-Tomás et al. (2009) showed that *Pantoea* and *Klebsiella* bacteria that are found in leaf-cutter ant fungus gardens fix nitrogen that supplements the ant diet, which is important for a strict herbivorous system. In addition to this, bacteria may also serve to fix nitrogen in leaf-cutter ant guts (Sapountzis et al., 2015). Nevertheless, the functional role of most garden bacteria remains unknown.

**FIGURE 1:**
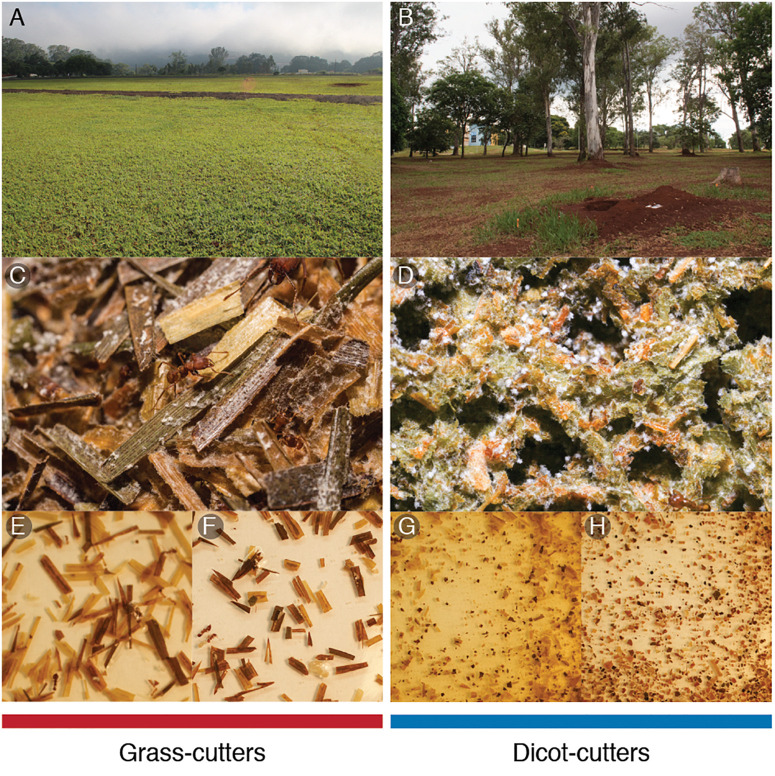
Grass- and dicot-cutter ants differ in the niches that they occupy, and the way that they cut and process leaf material. Field sites in **(A)** Botucatu, SP and **(B)** Ribeirão Preto, SP, Brazil. Fungus gardens of **(C)** grass- and **(D)** dicot-cutter ants. Visual inspection of leaf material from leaf-cutter ant fungus gardens demonstrates the degree of trituration that the different ants complete, with grass-cutters leaving the leaf material more intact (**E** – *A. bisphaerica* and **F** – *A. capiguara*), while dicot-cutters triturate to the point of producing unrecognizable leaf fragments (**G** – *A. laevigata* and **H** – *A. sexdens*).

While most leaf-cutter ants use dicots, three species of *Atta* are specialized on cutting grass, and another three species cut both grasses and dicots (Fowler et al., 1986). All previous studies on the microbial community in leaf-cutter ant fungus gardens have been focused on dicot-cutting ants, likely because dicot-cutters are more common and grass-cutter ants are notoriously difficult to maintain in the lab (Nagamoto et al., 2009). In this study, we compare the bacterial communities of fungus gardens from ants that cut grass and dicots (Figure 1). Given that grasses and dicots differ in terms of the cell wall composition, plant defense compounds (Wetterer, 1994; Mariaca et al., 1997) and nutrient availability (Mattson, 1980; Winkler and Herbst, 2004), we hypothesize that the bacterial community in these fungus gardens will differ in terms of community composition and functional capacity, in response to the different composition of the substrates the ants incorporate into their gardens. To address this, we collected fungus gardens from grass- and dicot-cutter ants and obtained their bacterial community metagenomes using Illumina sequencing. We analyzed the bacterial community in terms of its taxonomic composition, and its functional capacity. We also conducted analyses on the fungus gardens to determine their plant composition, their nutritional composition, and their plant defense compound contents.

## Materials and Methods

### Collection of Fungus Garden

Fungus gardens were collected on the campuses of the University of São Paulo (USP) in Ribeirão Preto, SP, Brazil and the São Paulo State University (UNESP) in Botucatu, SP, Brazil (Table 1). We collected fungus gardens from four species of *Atta* leaf-cutter ants: *A. bisphaerica* and *A. capiguara*, which are both described as grass-cutters, *A. laevigata*, which is described as a grass and dicot-cutter, and *A. sexdens*, which is described as a dicot-cutter (Fowler et al., 1986).

**TABLE 1:** Summary of collection details for leaf-cutter ant colonies used in this study.

Leaf-cutter ant colony	Substrate niche	IMG genome number	Collection date	Latitude	Longitude
*A. bisphaerica* 1	Grass	3300013023	1-Feb-15	S22°50′47.7″	W48°26′0.9″
*A. bisphaerica* 2	Grass	3300013025	3-Feb-15	S22°50′48.4″	W48°26′1.4″
*A. bisphaerica* 3	Grass	3300013022	3-Feb-15	S22°50′48.4″	W48°26′2.3″
*A. capiguara* 1	Grass	3300012994	2-Feb-15	S22°54′32.1″	W48°18′28.7″
*A. capiguara* 2	Grass	3300012996	3-Feb-15	S22°50′47.2″	W48°26′1.3″
*A. capiguara* 3	Grass	3300012997	3-Feb-15	S22°50′47.6″	W48°26′1.2″
*A. laevigata* 1	Dicot*	3300013000	20-Jan-15	S21°9′55.5″	W47°50′51.3″
*A. laevigata* 2	Dicot*	3300012995	17-Jan-15	S21°10′3″	W47°50′47″
*A. laevigata* 3	Dicot*	3300012998	19-Jan-15	S21°9′56.8″	W47°50′52.7″
*A. sexdens* 1	Dicot	3300012999	30-Jan-15	S21°9′50″	W47°51′26.9″
*A. sexdens* 2	Dicot	3300013002	30-Jan-15	S21°9′53.4″	W47°51′10.5″
*A. sexdens* 3	Dicot	3300013001	31-Jan-15	S21°10′2″	W47°51′5″

*While A. laevigata has been described as a grass/dicot-cutter ant (Fowler et al., 1986), due to its leaf-processing behavior and fungus garden plant composition observed in this study, we consider it a dicot-cutter.

For each species we collected from three independent colonies. To collect the fungus gardens, we identified the ant species by worker morphology then followed the entrance tunnel by digging until we found a fungus garden. Care was taken to expose fungus garden chambers from the side, to avoid damaging the garden with digging tools and avoid contamination with surrounding soil. Fungus gardens were transported to the laboratory and aseptically transferred into 50 mL conical tubes. When we sampled multiple chambers from one colony, they were always adjacent to each other and were superficially similar in appearance, texture and odor. The majority of worker ants were removed from the fungus garden material before being transferred to the tubes. In order to further reduce the chance of soil contamination, only intact fungus garden from the central region of the fungal mass was included in the tubes. Once filled, the tubes were frozen in liquid nitrogen, and stored at −80°C. At least four 50 mL conical tubes were filled from each colony. For each colony, two tubes were combined and used for metagenomics, one tube was used for iron content measurements, and one tube was used for gas chromatography (Supplementary Material). The decision to only sample from the central region of the fungus garden was taken for a few reasons. As mentioned above, it was important to reduce the chance of contamination from soil bacteria. Furthermore, earlier work by Aylward et al. (2012a) demonstrated that the bacterial community is conserved through layers of the fungus garden. The middle layer was also of most importance to us since it is where most of the gongylidia are present, where developing ants obtain the majority of their nutrition, and where we expect the greatest impact on ant fitness. As such, and due to the tremendous resources necessary to complete community metagenomic sequencing, we focused on this area.

### DNA Extraction

To target the bacteria in the fungus gardens, DNA was extracted by first using a differential centrifugation method (Aylward et al., 2012a). PBS buffer with 1% Tween 80 was added to the tubes and they were vortexed for 30 min. They were then kept at 4°C for 30 min so that large particles would settle. The liquid portion was decanted and passed through a 40 μm filter. The filtrate was centrifuged for 30 min at 4°C at 4300 rpm (Beckman Coulter X-14R centrifuge with an SX4750 swinging bucket), after which a bacterial cell pellet was formed and the liquid was removed. This process was repeated with the original fungus garden tube to wash off any remaining bacterial cells from the leaf material. DNA was extracted from the cell pellet using the Qiagen Plant DNA Extraction Maxi Kit (Qiagen, Hilden, Germany). The remaining leaf material from the fungus gardens was photographed after the differential centrifugation, to demonstrate the difference in leaf material consistency (Figure 1).

### DNA Sequencing and Assembly

All metagenomic sequencing was conducted at the Joint Genome Institute (JGI) in Walnut Creek, CA. Since some DNA concentrations were too low for standard library prep, a low-input prep was completed for all of the samples. For each sample, 10 ng of DNA was sheared to 300 bp using the Covaris LE220 (Covaris) and size selected using SPRI beads (Beckman Coulter). The fragments were treated with end-repair, A-tailing, and ligation of Illumina compatible adapters (IDT, Inc.) using the KAPA-Illumina library creation kit (KAPA Biosystems) and 10 cycles of PCR were used to enrich for the final library. The prepared libraries were quantified using KAPA Biosystems next-generation sequencing library qPCR kit and run on a Roche LightCycler 480 real-time PCR instrument. The quantified libraries were then prepared for sequencing on the Illumina HiSeq sequencing platform utilizing a TruSeq Rapid paired-end cluster kit, v4. Sequencing of the flowcell was performed on the Illumina HiSeq2500 sequencer using HiSeq TruSeq SBS sequencing kits, following a 2 × 150 indexed run recipe. BBDuk adapter trimming (Bushnell, 2017) was used to remove known Illumina adapters. The reads were then processed using BBDuk filtering and trimming, where quality values were less than 12. We discarded read pairs that fit certain criteria: those containing more than three ambiguous bases, or quality scores (before trimming) averaging less than three over the read, or length under 51 bp after trimming, as well as reads matching Illumina artifact, spike-ins or phiX. Trimmed, screened, paired-end Illumina reads were assembled using the megahit assembler using with the “–k-list 23,43,63,83,103,123” option. Functional annotation and taxonomic classification were performed using the Integrated Microbial Genomes (IMG) pipeline (Chen et al., 2018).

### Plant Genus Richness

To determine the richness of plant substrate integrated in the fungus gardens of the ants, we used JGI’s IMG database “find gene” function to retrieve all genes annotated as *MatK* from the dataset. *MatK* is a widely used chloroplast plant DNA barcode (Hollingsworth et al., 2011). Retrieved *MatK* sequences for each metagenome were identified using BLASTn (Altschul et al., 1990). The best match for each sequence was identified first to the species level (where all matches were at least 98%) but to ensure consistent and reliable certainty with the identified plants, we identified all sequences to the genus level. Because most of the plant biomass was removed from samples before DNA extraction only presence/absence of genera were considered, not abundance.

### Bacterial Taxonomic Analysis

Relative abundance of bacterial taxa (classes and genera) were determined using MATAM (Pericard et al., 2018). Reads originated from 16s genes were identified and assembled into contigs, and we set the coverage threshold to be 500×. We also used MATAM for taxonomic assignment using the “perform taxonomic assignment” option. This calls an RDP Classifier (Wang et al., 2007), which is a naive Bayesian classifier with the default training model “16srrna.” MATAM also reports the coverage of each contig to be used for the calculation of relative abundance; here we only kept those with coverage above 1 as a quality control, excluded contigs that could not be identified, and collapsed taxa that represented less than 1% of the community into one category. We used the relative abundances of each phylum and genus to run a non-metric multidimensional analysis (NMDS) using a Bray-Curtis dissimilarity index with the vegan package in the R statistical programming environment (Oksanen et al., 2013; R Core Team, 2013). Relative abundance was calculated as the proportion of each bacterial genus or class compared to the total quantity of bacteria in the sample (McKnight et al., 2018). Also using the vegan package, we used ANOSIM and PERMANOVA to determine if groups (grass-cutters vs. dicot-cutters) or species (*A. sexdens, A. laevigata, A. capiguara*, and *A. bisphaerica*) were significantly different. We also used ANOSIM and PERMANOVA to compare bacterial communities based on species within substrate groups. Finally, using the vegan package we calculated the Shannon diversity index to compare the diversity of each sample on the basis of the bacterial genera present. We tested whether dicot-cutter ant fungus gardens have a more diverse bacterial community by comparing the Shannon diversity of the genera, and also comparing genus richness, both using two-sample *t*-tests in the R statistical programming environment. To test whether specific genera have significantly different relative abundances between grass- and dicot-cutter ant fungus gardens, we used DESeq2 in the R statistical programming environment (Love et al., 2014).

### Bacterial Functional Analysis

In order to make functional comparisons of the bacteria in grass- and dicot-cutter fungus gardens, we used the Kyoto Encyclopedia of Genes and Genomes (KEGG) annotations of the metagenomes through IMG’s KEGG Orthology (KO) pipeline, which is part of JGI’s standard operating procedure (Huntemann et al., 2016). Briefly, genes were associated with KO terms (Kanehisa et al., 2014) based on USEARCH 6.0.294 results (Edgar, 2010) and were filtered for minimum identity matches and gene sequence coverage. For an overall comparison of functional differences between the fungus gardens, we used the same ordination and statistical methods as for bacterial genus relative abundance. As with genus group differences, we used DESeq2 to determine what genes are significantly enriched between grass- and dicot-cutter ant fungus gardens. Since DESeq2 requires inputs to be integers, we used number of gene copies per million genes in the metagenomes as our input (Alneberg et al., 2014).

### Iron Content

Separate 50 mL tubes of fungus garden material, from the same colonies as above, were used for determination of iron content. All ants were removed from fungus gardens then the remaining fungus garden material (triturated plants covered in fungus) was analyzed at the UW Soil and Forage Lab in Marshfield, WI, using standard methods. Approximately 0.5 g of dried and ground fungus garden material was weighed out into a folin digestion tube. The material was then digested in 5 mL of concentrated nitric acid (67–70%), and heated to 120°C for 4 h. Next, 1 mL of hydrogen peroxide (30%) was added, and the samples were heated for a further 20 min before being diluted and analyzed by inductively coupled plasma optical emission spectroscopy (ICP-OES) (Fassel and Kniseley, 1974).

## Results

### Metagenomic Statistics

A summary of metagenome statistics is presented in Table 2. A total of 5.316 Gbp of assembled sequence data was produced in this study, with an average of 443 Mbp per metagenome. The smallest metagenome was from the grass-cutter colony *A. capiguara* 1 at 148.7 Mbp, and the largest metagenome was from the dicot-cutter colony *A. sexdens* 2 at 812.9 Mbp. Maximum scaffold lengths ranged from 61.96 to 701.42 Kbp, with an average maximum scaffold length of 266.6 Kbp. Between 91.63 and 99.31% of reads were aligned.

**TABLE 2:** Metagenome sequencing statistics for leaf-cutter ant fungus gardens.

Leaf-cutter ant colony	Scaffold total	Scaffold sequence total (Mbp)	Main genome scaffold N/L50	Main genome scaffold N/L90	Max scaffold length (Kbp)	Scaffolds > 50 Kbp	Aligned reads	Protein coding genes
*A. bisphaerica* 1	628724	390.8	122506/740	467228/298	249.52	93 (2.07%)	163185122 (98.76%)	607 042 (99.39%)
*A. bisphaerica* 2	939707	630.9	148370/926	680177/292	253.54	69 (0.88%)	148406252 (96.49%)	910 609 (99.61%)
*A. bisphaerica* 3	285649	186.0	29401/972	244722/247	187.50	49 (1.90%)	167169016 (98.76%)	358 547 (98.19%)
*A. capiguara* 1	205334	148.7	16608/1403	178745/247	273.83	37 (2.49%)	199009346 (99.31%)	272 096 (98.99%)
*A. capiguara* 2	345332	261.2	35330/1420	303130/247	180.27	34 (1.06%)	204772230 (98.31%)	456 916 (98.70%)
*A. capiguara* 3	573737	359.5	83958/790	508567/247	135.51	13 (0.29%)	203079026 (98.77%)	644 865 (98.79%)
*A. laevigata* 1	853367	517.5	178678/686	645038/301	274.98	87 (1.53%)	178461364 (96.65%)	871 330 (99.42%)
*A. laevigata* 2	295365	189.2	32897/990	266928/247	252.44	92 (4.23%)	189659024 (96.85%)	332 737 (96.95%)
*A. laevigata* 3	601593	546.3	74824/1744	398992/340	241.71	17 (0.30%)	209535750 (96.06%)	722 718 (99.01%)
*A. sexdens* 1	674609	708.4	48220/3118	412923/341	701.43	167 (2.40%)	156148622 (97.92%)	822 403 (99.46%)
*A. sexdens* 2	857038	812.9	65552/2346	548662/328	61.96	17 (0.11%)	150809208 (95.32%)	1 088 719 (99.51%)
*A. sexdens* 3	1006806	564.7	221493/614	772141/277	386.55	68 (1.17%)	186976430 (91.63%)	1 029 784 (99.20%)

### Bacterial Taxonomic Analysis

**FIGURE 2:**
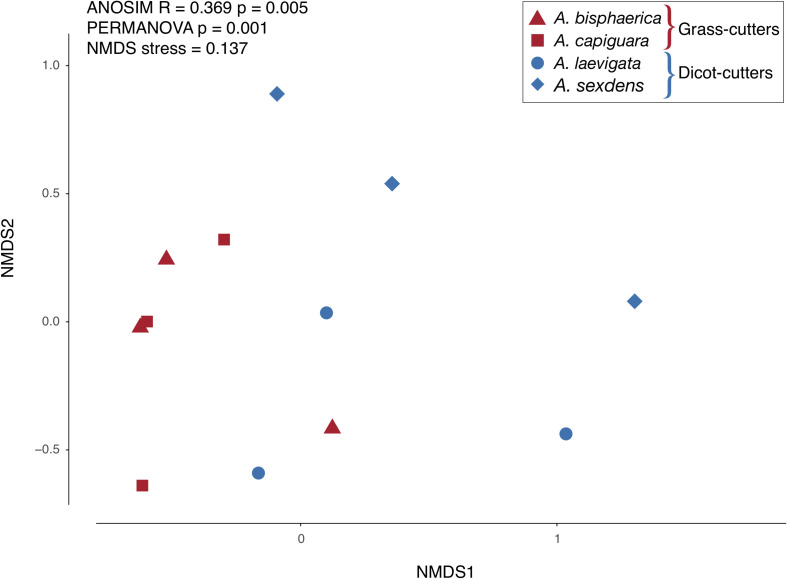
NMDS plot of the relative abundance of bacterial genera in fungus gardens of grass- and dicot-cutter ants.

**FIGURE 3:**
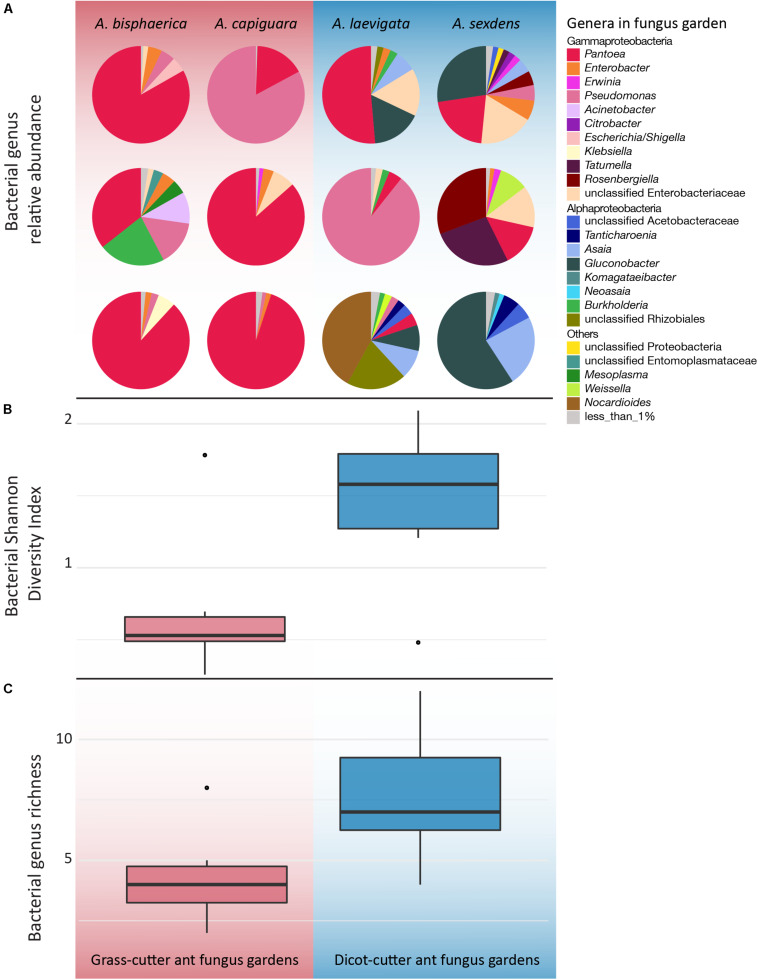
Genus-level bacterial community analysis of leaf-cutter ant fungus gardens from grass- and dicot-cutter ants, demonstrating that dicot-cutter, ant fungus gardens have a higher diversity of bacteria. All data are based on 16S sequences extracted from the metagenomes using MATAM, and are relative abundances. **(A)** Pie charts showing proportions of different bacterial genera in the fungus gardens. **(B)** Shannon diversity index of bacterial genera (*t* = -2.3332, *df* = 10, *p* = 0.0209). **(C)** Bacterial genus richness (*t* = -2.3063, *df* = 10, *p* = 0.02189). **(B,C)** Only include genera that consist of more than 1% of the total relative abundance.

Overall, while there is some overlap in the NMDS plot, *Atta* spp. fungus garden bacterial communities differed from each other, depending on if the ants were grass- or dicot-cutters (ANOSIM *R* = 0.369, *p* = 0.005, PERMANOVA *p* = 0.001) (Figure 2). The same analysis based on species (both across all samples and within substrate-specialized groups) did not show a significant difference. All fungus garden bacterial communities were dominated by Proteobacteria. Grass-cutter ant fungus gardens were comprised mostly of Gammaproteobacteria (94%), and while their proportion was still high, it was lower in dicot-cutter ant fungus gardens (54%). Alphaproteobacteria were also a dominant class in dicot-cutter ant fungus gardens where they were 34% of the average bacterial community. The most relatively abundant genus in all the fungus gardens was *Pantoea*, at 67 and 16% of the grass- and dicot-cutter ant fungus gardens, respectively. Other abundant genera were *Pseudomonas* at 18 and 16% of the grass- and dicot-cutter ant fungus gardens, respectively, and *Gluconobacter*, which was 19% of the bacterial community in dicot-cutter ant fungus gardens, but was not found at all in grass-cutter ant fungus gardens. *Burkholderia* and *Enterobacter* were found in both fungus gardens at an average proportion of 2% of the bacterial community (Figure 3A). Both grass- and dicot-cutter ant fungus gardens contained bacterial genera that were unique to the their respective groups, but there were more of these substrate-specific bacteria in the dicot-cutter ant fungus gardens, and this contributed to the higher overall diversity of bacteria in them (Shannon diversity index of 1.46) compared to the grass-cutter ant fungus gardens (Shannon diversity index of 0.71) (*t* = -2.3332, *df* = 10, *p* = 0.0209) (Figure 3B). This is also reflected in the mean genus richness, which were 7.7 and 4.3 genera in the dicot- and grass-cutter ant fungus gardens, respectively (*t* = -2.3063, *df* = 10, *p* = 0.02189) (Figure 3C). Despite these patterns, DESeq2 analysis did not find significant differences between particular high-abundant genera between the two types of fungus gardens, likely due to low power in the analysis.

### Bacterial Functional Analysis

**FIGURE 4:**
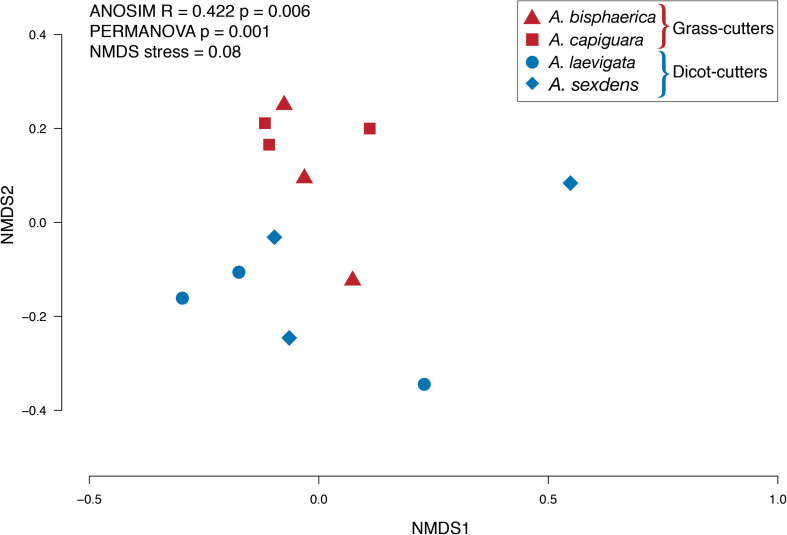
NMDS plot of KO functional genes from grass- and dicot-cutter ant fungus gardens.

**FIGURE 5:**
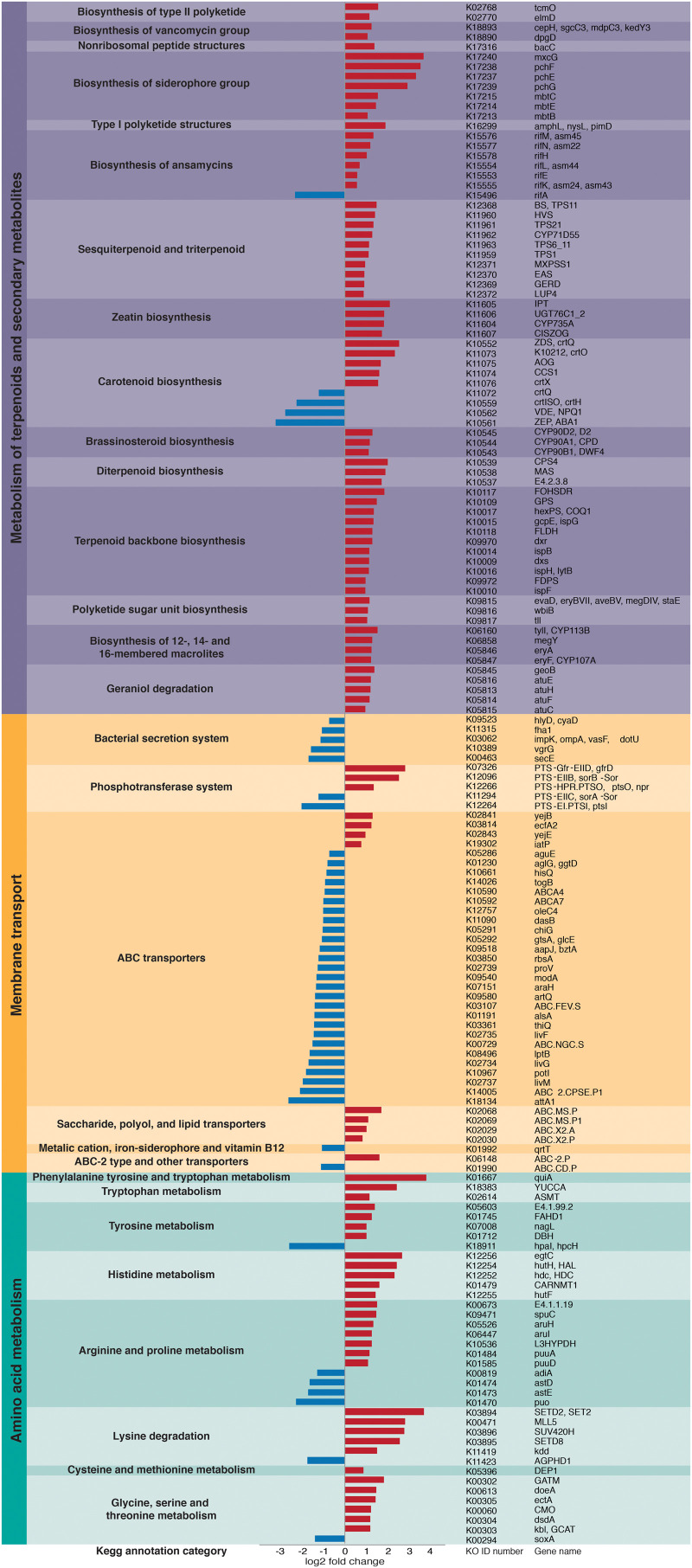
Particular groups of genes are enriched in either the grass- or dicot-cutter ant fungus gardens. Bars extending to the left (blue) represent genes that are significantly more abundant in dicot-cutter ant fungus gardens and bars extending to the right (red) represent genes that are significantly more abundant in grass-cutter ant fungus gardens.

Overall, we found significant differences in the predicted bacterial community functional profiles between grass- and dicot-cutter ant fungus gardens (ANOSIM *R* = 0.422, *p* = 0.006, PERMANOVA *p* = 0.001) (Figure 4). All individual bacterial genes that were significantly different between grass- and dicot-cutter ant fungus gardens are listed in Supplementary Table 1. In total, 514 predicted bacterial genes were significantly enriched, with 313 and 201 genes significantly enriched in grass- and dicot-cutter ant gardens, respectively (Supplementary Table 2 and Supplementary Figures 4–6). Grass-cutter ant fungus gardens were enriched for amino acid biosynthesis genes for phenylalanine, tryptophan, tyrosine, histidine, arginine, lysine, cysteine, methionine, glycine, serine and threonine. They were also significantly enriched in terpenoid and siderophore biosynthesis genes (Figure 5) and had a significantly higher relative abundance of a gene in the nitrogen fixation pathway, nitrogenase molybdenum-iron protein beta chain (Supplementary Table 2). Dicot-cutter ant fungus gardens were particularly enriched in membrane transport genes (Figure 5).

### Plant Taxonomy and Consistency

The incorporated plant material was markedly different in consistency between the fungus gardens. *Atta bisphaerica* and *A. capiguara* gardens both contained material that was clearly grass, which was not triturated (Figure 1). In contrast, the leaf material in the fungus gardens of *A. laevigata* and *A. sexdens* was triturated to the point of being unrecognizable as plant material (Figure 1). We detected 68 plant species based on the *MatK* gene query in the metagenomes, from 40 genera and 15 families. The fungus gardens of dicot-cutter ants had a significantly higher richness of plant genera than those of grass-cutter ants (ANOVA F = 9.14, *p* = 0.0128). As expected, the grass-cutter ant fungus gardens all contained grass (*Paspalum*, Poaceae). The dicot-cutter ant fungus gardens contained more genera and families of plants, which were mostly dicots, but three of these fungus gardens also contained some grass (Table 3). Although *A. laevigata* has been described in the literature as a grass and dicot-cutter, here we consider it a dicot-cutter for several reasons: the fungus garden consistency and amount of leaf trituration were like those of other dicot-cutter ants (Figure 1), the *MatK* data show that grass was not found in the fungus gardens of *A. laevigata* colony 2 (Table 3), and finally, the colonies used in the study were observed to be cutting mostly dicots even though all *A. laevigata* colonies were found in areas where both grasses and dicots were available (personal observation, Figure 1). As a result, we would expect that the bacterial community would be exposed to conditions most similar to other dicot-cutter ant fungus gardens.

**TABLE 3:** Plant genera detected in each fungus garden sample using *MatK* gene.

Sample	Family	Genus	Species *MatK* match%
*A. bisphaerica* 1	Fabaceae	*Chamaecrista*	99.3
	Poaceae	*Paspalum*	99.6
	Polygalaceae	*Polygala*	99.3
*A. bisphaerica* 2	Poaceae	*Paspalum*	99.4
*A. bisphaerica* 3	Fabaceae	*Chamaecrista*	99.3
	Fabaceae	*Zornia*	100
	Poaceae	*Paspalum*	99.5
	Polygalaceae	*Polygala*	99.0
*A. capiguara* 1	Poaceae	*Paspalum*	99.7
*A. capiguara* 2	Poaceae	*Paspalum*	99.6
*A. capiguara* 3	Fabaceae	*Chamaecrista*	99.3
	Poaceae	*Paspalum*	99.6
*A. laevigata* 1	Fabaceae	*Pterogyne*	99.4
	Myrtaceae	*Eucalyptus*	99.9
	Poaceae	*Paspalum*	99.5
	Poaceae	*Urochloa*	100
*A. laevigata* 2	Asteraceae	*Rensonia*	99.6
	Fabaceae	*Centrolobium*	98.2
	Fabaceae	*Schizolobium*	100
*A. laevigata* 3	Anacardiaceae	*Pachycormus*	98.6
	Asteraceae	*Kingianthus*	95.4
	Asteraceae	*Podanthus*	99.5
	Fabaceae	*Desmodium*	99.8
	Fabaceae	*Leucaena*	100
	Myrtaceae	*Eucalyptus*	99.8
	Poaceae	*Paspalum*	99.9
*A. sexdens* 1	Anacardiaceae	*Loxopterygium*	98.4
	Asteraceae	*Cymophora*	98.5
	Bignoniaceae	*Tabebuia*	98.1
	Fabaceae	*Andira*	98.6
	Fabaceae	*Batesia*	98.8
	Fabaceae	*Bussea*	100
	Fabaceae	*Libidibia*	99.8
	Fabaceae	*Pterogyne*	100
	Fabaceae	*Tipuana*	99.9
	Malvaceae	*Pachira*	100
	Myrtaceae	*Eucalyptus*	99.6
	Myrtaceae	*Eugenia*	99.8
	Poaceae	*Scutachne*	98.4
	Rubiaceae	*Genipa*	99.2
	Solanaceae	*Lycianthes*	100
*A. sexdens* 2	Bignoniaceae	*Tabebuia*	98.3
	Combretaceae	*Lumnitzera*	93.4
	Fabaceae	*Centrolobium*	98.2
	Fabaceae	*Pterogyne*	99.3
	Fabaceae	*Tipuana*	100
	Lecythidaceae	*Careya*	94.0
	Santalaceae	*Phoradendron*	99.6
*A. sexdens* 3	Asteraceae	*Echinacea*	99.3
	Asteraceae	*Eclipta*	100
	Asteraceae	*Perymeniopsis*	99.8
	Asteraceae	*Synedrella*	100
	Commelinaceae	*Commelina*	100
	Commelinaceae	*Murdannia*	92.0
	Fabaceae	*Desmodium*	99.8
	Fabaceae	*Leucaena*	100
	Malvaceae	*Sida*	99.7
	Myrtaceae	*Eucalyptus*	98.6
	Phyllanthaceae	*Phyllanthus*	100
	Rubiaceae	*Genipa*	100
	Solanaceae	*Acnistus*	99.4

### Iron Content

**FIGURE 6:**
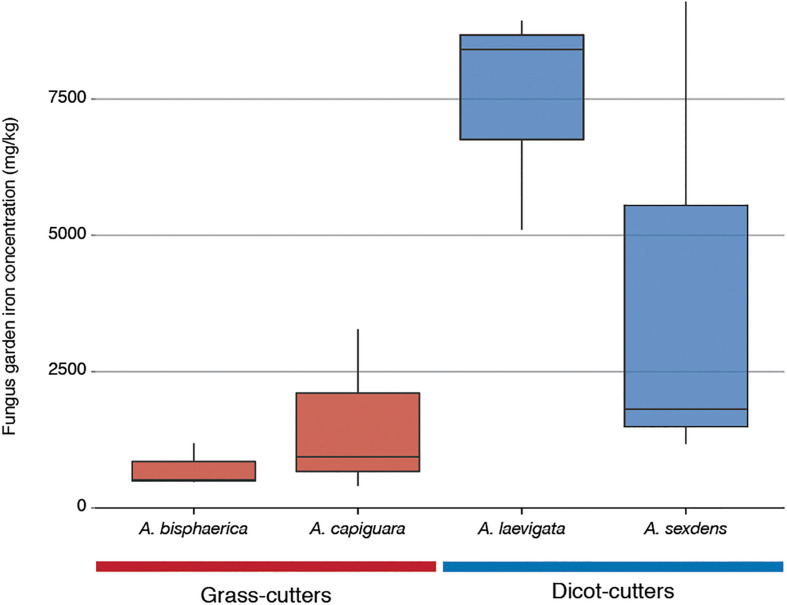
Iron content of fungus gardens from this study as measured by inductively coupled plasma optical emission spectroscopy.

The iron content of the fungus gardens is displayed in Figure 6. This represents the iron in the fungus garden, originated from plants. The grass-cutter ant fungus gardens have lower amounts of iron than the dicot-cutter ant fungus, but this difference is not significant due to the high variability between *A. sexdens* gardens.

## Discussion

We can better understand animal diet specialization and transitions to novel substrates by understanding how microbial symbiont communities change in relation to transitions in host substrate use. *Atta* ants provide a relatively unique opportunity to examine a group of closely related herbivores that have transitioned from specialization on dicots to grasses. These plants differ in terms of their cell wall composition, nutrient density and defense compounds. Here, using metagenomic sequencing, we examine this transition in the bacterial community in the fungus gardens of ants that are specialized on these different substrates. The results of this study demonstrate that bacterial communities differ in composition and functional capacity, depending on the type of substrate ants incorporate into their gardens. These differences suggest that the bacteria might play a role in mediating the relationship between leaf-cutter ant colonies and the plants they consume.

While the grass- and dicot-cutter ant fungus gardens differed significantly, it is interesting to note that there is some overlap between the *A. laevigata* fungus garden bacterial community with that of the grass-cutters *A. bisphaerica* and *A. capiguara* (Figure 2). This may be a reflection of *A. laevigata* as a transitional species in terms of substrate choice. These ants do incorporate some grass into their fungus gardens (Table 3) so it should not be surprising that there is more overlap between the bacterial communities in these fungus gardens and the grass-cutter ant fungus gardens. Even so, we maintain that, both in terms of the metagenomes and fungus garden characteristics, *A. laevigata* is best categorized as a dicot-cutter ant species.

If bacteria in fungus gardens are responsible for the breakdown of recalcitrant plant biomass, which is found in plant cell walls, we would expect bacterial communities in the two ant groups examined would be differentially enriched in genes necessary for plant biomass breakdown. Grass cell walls contain (1→3),(1→4)-β-D-glucan chains and silica, neither of which are present in dicots (Popper and Tuohy, 2010). In other systems specialized on grass biomass breakdown, the microbes responsible for this produce specialized enzymes (King et al., 2011) and have genomes that are adapted for this function (Wolfe et al., 2012). In this system, between the bacterial communities in grass- and dicot-cutter ant fungus gardens, we do not identify a significant difference in the relative abundance of genes responsible for plant biomass degradation, namely glycoside hydrolases, carbohydrate esterases, carbohydrate binding molecules, polysaccharide lyases, and glycosyl transferases (Supplementary Table 1). Thus, we conclude that garden bacteria do not respond to changes in cell wall structure between grasses and dicots. Instead, we expect that the genome or gene expression in the fungus from these two systems would show differences, especially since the fungus is the primary degrader of plant biomass in leaf-cutter ant fungus gardens (Nagamoto et al., 2011; Aylward et al., 2013; Grell et al., 2013; Khadempour et al., 2016).

Leaf-cutter ants, in general, cut an exceptionally broad diversity of plants (Mayhé-Nunes and Jaffe, 1998; Solomon, 2007) and thus, have the potential to encounter a myriad of plant defense compounds that are toxic to themselves and their fungal cultivar. Leaf-cutter ant genomes are not enriched in gene families for plant defense compound detoxification (Rane et al., 2016), so they must reduce the intake of these chemicals in other ways, many of which involve ant behavior, such as foraging preferences and leaf processing (Hubbell et al., 1984; Howard, 1988; Wirth et al., 1997; Hart and Ratnieks, 2000; Roschard and Roces, 2003). Nevertheless, some quantity of volatiles can make their way into the gardens (Supplementary Methods and Supplementary Figure 2).

In order to mitigate deleterious effects of plant defense compounds, we expect that *L. gongylophorus* would produce enzymes to degrade them. Indeed, work by De Fine Licht et al. (2013) implicates an important role for fungal cultivar-derived laccases in detoxifying these compounds. However, bacteria in the garden may also mediate effects of plant defense compounds, especially those that are toxic to the fungus, or that the fungus does not have the capacity to detoxify. Some evidence that bacteria found in dicot-cutter ant fungus gardens are better equipped to contend with toxic plant compounds is the higher abundance of membrane transport genes, especially ABC transporters in dicot-cutter ant fungus gardens (Figure 5), as these are known to be important in responding to toxins (Putman et al., 2000; Glavinas et al., 2004). The bacterial community contains the genes necessary for plant defense compound remediation, including many cytochrome P450s, gluthione S-transferases, and other genes involved in xenobiotic degradation (Supplementary Table 1), and aromatic compound degradation (Supplementary Figures 4, 5), but they are not consistently enriched in the dicot-cutter ant fungus gardens. Since dicot-cutter ants incorporate a higher diversity of plants into their gardens (Table 3), we expect that the diversity of bacteria would also be higher in these gardens, and that the bacteria would have a higher capacity for the degradation of defense compounds. While we did observe a greater diversity of bacteria in the dicot-cutter ant fungus gardens (Figure 3), we did not see a significant enrichment of plant defense compound degradation genes in these gardens (Figure 5 and Supplementary Table 1). However, we still cannot exclude the possibility that bacteria are taking part in this process. Since each dicot-cutter ant colony cuts a unique and diverse set of plants (Table 3), they potentially encounter unique and diverse plant chemistries, including plant defense compounds. If the bacterial community were to respond in a substrate-specific manner to different plant defense compounds, our analysis in this study would not reveal that. In addition, we sampled only the middle section of the fungus gardens, for reasons described in the section “Materials and Methods.” It is possible that we would see an enrichment for plant defense compound detoxification genes in the top of the fungus garden, where fresh plant material is deposited. To elucidate the role of bacteria in plant defense compound remediation, closely controlled experiments with particular defense compounds of interest applied to bacterial cultures and to fungus gardens would be necessary, and have been pursued as a follow-up to this study (Francoeur et al., 2020).

Pinto-Tomás et al. (2009) established that *Pantoea* and *Klebsiella* bacteria in Central American leaf-cutter ant fungus gardens supplement the ant diet through nitrogen fixation. Many herbivores supplement their diets through bacterial nitrogen fixation because the plants they consume do not supply enough (Douglas, 2009; Hansen and Moran, 2013). Grasses are especially low in nitrogen (Mattson, 1980; Winkler and Herbst, 2004), so we predict that grass-cutter ant fungus gardens would be enriched in nitrogen-fixing bacteria with a corresponding enrichment of nitrogen-fixing genes. Here we show that a nitrogenase molybdenum-iron protein beta chain gene is significantly more abundant in grass-cutter ant fungus gardens (Supplementary Table 1). Other genes that are related to nutrient acquisition are also significantly more abundant in these gardens (Figure 5), such as genes in amino acid metabolism pathways. While it has been shown that nitrogen fixed by bacteria is incorporated into the bodies of ants (Pinto-Tomás et al., 2009), animals cannot simply absorb nitrogen as ammonium or nitrate, they require it to either be in the form of amino acids or other organic nitrogen-containing compounds (White, 1993). The enrichment of arginine biosynthesis genes is of particular interest since the genome of *Atta* is deficient in genes in this pathway (Suen et al., 2011b). While a transcriptome study of *L. gongylophorus* demonstrated that the cultivar has the genes necessary for arginine biosynthesis (Licht et al., 2014), the bacteria could supplement this process.

Other categories of genes enriched in the grass-cutter ant fungus garden bacteria are those involved in metabolism of terpenoids and other secondary metabolites, especially their biosynthesis. Grass-cutter ant fungus gardens are significantly enriched in 67 of these genes. This list includes seven siderophores, which are responsible for iron acquisition (Crosa, 1989; Winkelmann, 2002). Siderophores are costly to produce so enrichment of these genes suggests that iron acquisition is important in this system. The grass-cutter ant fungus gardens examined in this study contained lower amounts of iron than the dicot-cutter ant fungus gardens (Figure 6). Since iron is an important cofactor in cells, and typically has low concentration in soils, it is a limiting resource for plants, and the organisms that feed on them (Briat et al., 2006). The movement of iron in the leaf-cutter ant fungus gardens provides an interesting and unexplored avenue for future studies.

Terpenoids are the most abundant secondary metabolites found in plants, and serve diverse roles (Langenheim, 1994; Gershenzon and Dudareva, 2007). The majority of research into the connection between plant terpenoids and animal-microbe symbioses are in regard to the detoxification of terpenes that would be deleterious to the animal host (Wang et al., 2012; Adams et al., 2013; Boone et al., 2013; Cheng et al., 2013; Raffa, 2013). However, not all terpenes are toxic to all organisms (Raffa, 2013), and in at least one instance they have been shown to supplement a herbivore’s diet after some modification by a gut bacterium (Berasategui et al., 2017). Dicots contain higher quantities of terpenoids (Wetterer, 1994; Mariaca et al., 1997). One possibility is that the bacteria in these fungus gardens are producing terpenes as a nutritional additive, especially in the grass-cutter ant fungus gardens where there are lower terpene inputs and these genes are enriched (Figure 5 and Supplementary Figure 2).

Overall, this study demonstrates that the bacterial community differs in both community composition and functional capacity between grass- and dicot-cutter ant fungus gardens. We argue that the difference in functional capacity of bacteria in the different gardens can be used by the fungus and, downstream of that, the ants in this system. As an extension of this, the bacteria may allow grass-cutter ants to utilize a lower quality substrate than their dicot-cutter counterparts. Of course, this is all based on metagenomic work, and has its limitations. First, the steps of transcription and translation lie between the number of gene copies and ultimate function. However, previous efforts to examine the bacterial community *in situ*, both through metaproteomics and metatranscriptomics, have not been fruitful, since the fungal biomass, transcripts, and proteins swamp out the bacterial portion (Khadempour et al., 2016; Moreira-Soto et al., 2017). Therefore, to date, this work provides the most comprehensive analysis of the bacterial community in leaf-cutter ant fungus gardens. Follow-up studies will focus on bacteria both in culture and in fungus gardens using more targeted approaches to either confirm or contradict the results of this study, which suggest that the bacteria in leaf-cutter ant fungus gardens may play a role in mediating the relationship between ants and the types of plants that they incorporate into their gardens.

## Data Availability Statement

The datasets generated for this study can be found in the online repositories. The names of the repository/repositories and accession number(s) can be found below: https://img.jgi.doe.gov/, 3300013023, 3300013025, 3300013022, 3300012994, 3300012996, 3300012997, 3300013000, 3300012995, 3300012998, 3300012999, 3300013002, and 3300013001.

## Author Contributions

LK and CC conceived of the project. LK, HF, and CC-S performed computational and statistical analysis. LK designed sampling methodology. LK, MD, and KK-R performed lab work. LK and NN performed field work. MP and CC provided resources and facilities. LK, HF, KK-R, and CC wrote the manuscript. All authors contributed to the article and approved the submitted version.

## Conflict of Interest

The authors declare that the research was conducted in the absence of any commercial or financial relationships that could be construed as a potential conflict of interest. The reviewer, PK declared a shared affiliation with one of the authors, NN, to the handling editor at the time of review.
