# Supplementary figures and images for: Metagenomics Reveals Diet-Specific Specialization of Bacterial Communities in Fungus Gardens of Grass- and Dicot-Cutter Ants
Source: Front Microbiol. 2020 Sep 24;11:570770. doi: 10.3389/fmicb.2020.570770 (PMC7541895; doi:10.3389/fmicb.2020.570770)

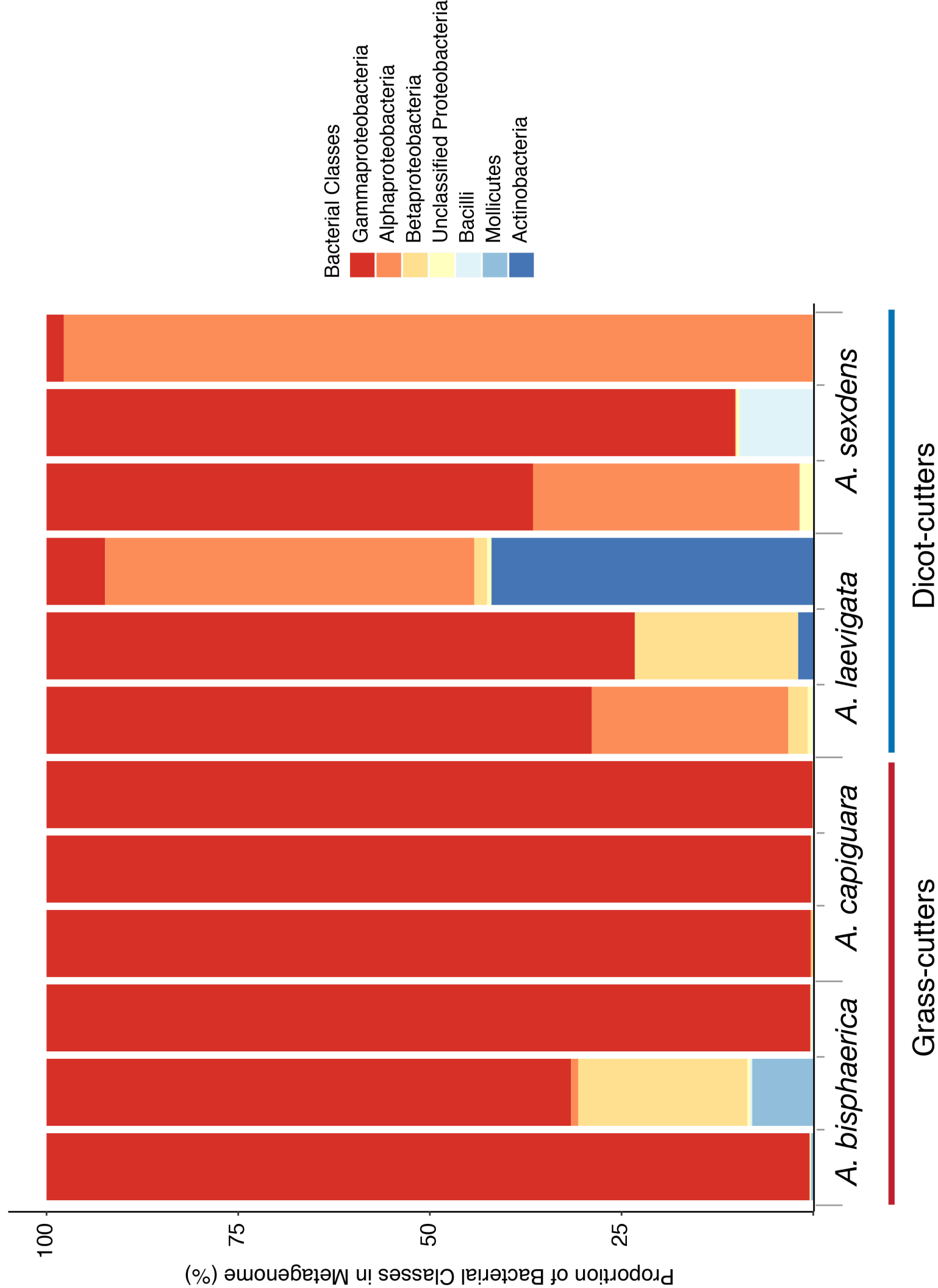

Supplement: Supplementary file 1 [file Image_1.PDF]

## Monoterpenoid biosynthesis

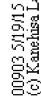

Supplement: Supplementary file 3 [file Image_3.PDF]

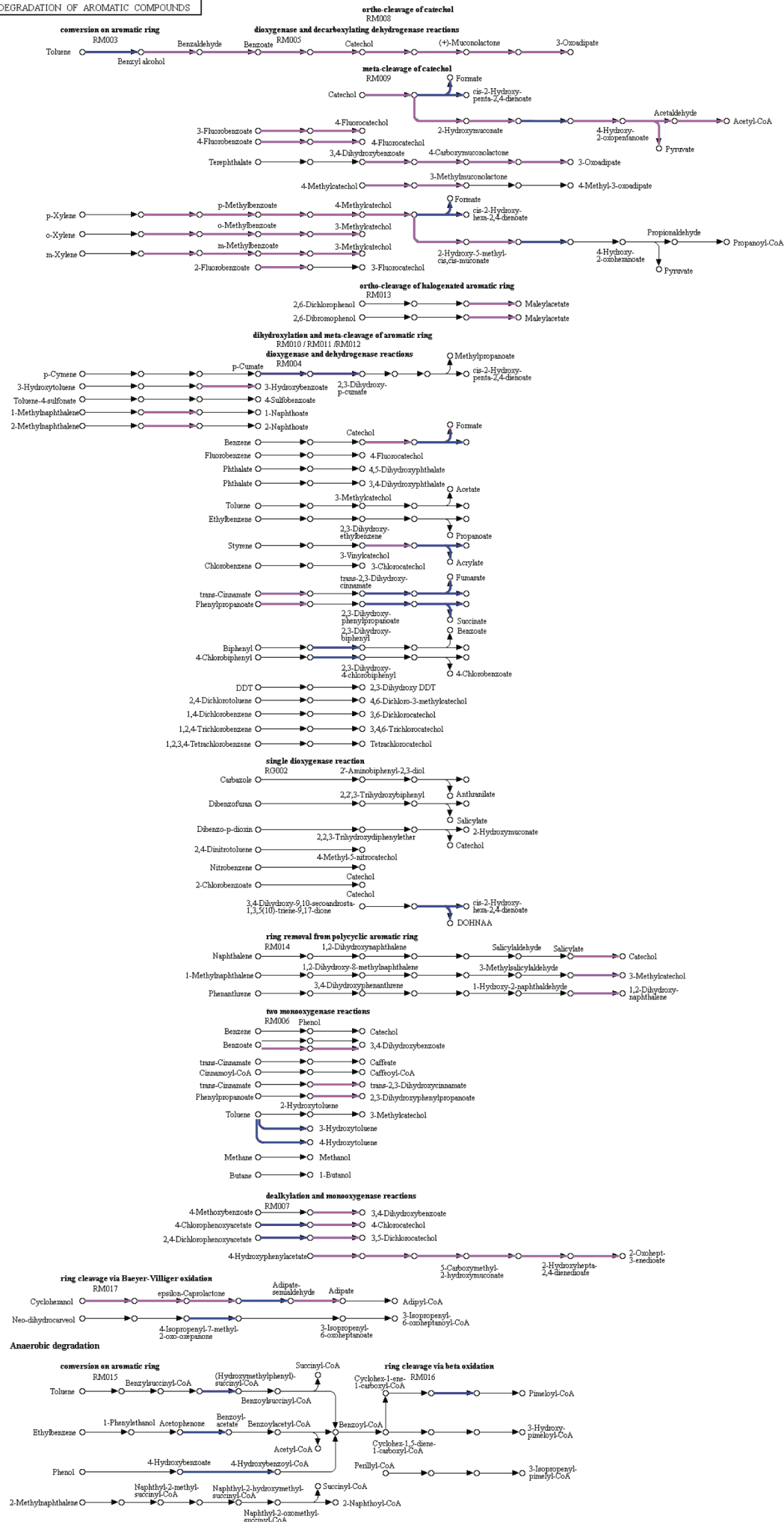

Supplement: Supplementary file 4 [file Image_4.PDF]

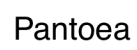

## Pantoea

Supplement: Supplementary file 5 [file Image_5.PDF]

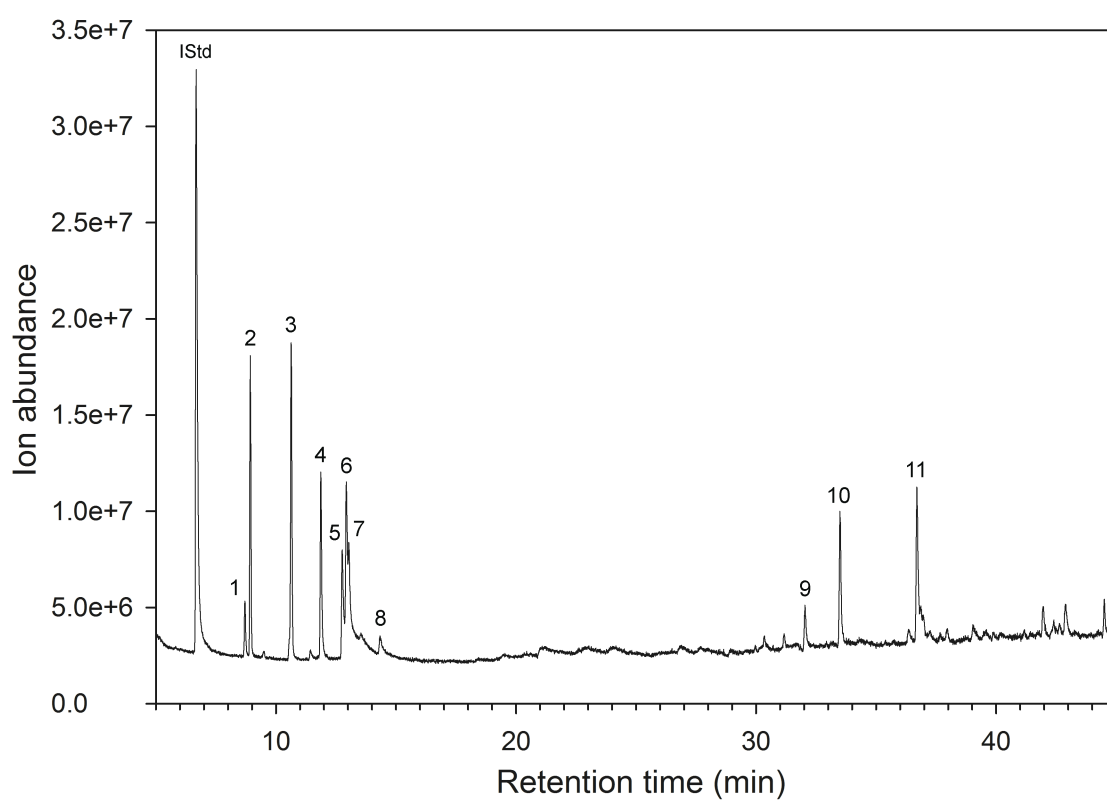

Supplement: Supplementary file 6 [file Image_6.PDF]
