# Supplementary material for: Metagenomics Reveals Diet-Specific Specialization of Bacterial Communities in Fungus Gardens of Grass- and Dicot-Cutter Ants
Source: Front Microbiol. 2020 Sep 24;11:570770. doi: 10.3389/fmicb.2020.570770 (PMC7541895; doi:10.3389/fmicb.2020.570770)
Supplement: Supplementary file 2 [file Image_2.PDF]

ABC TRANSPORTERS

Prokaryotic-type ABC transporters

E

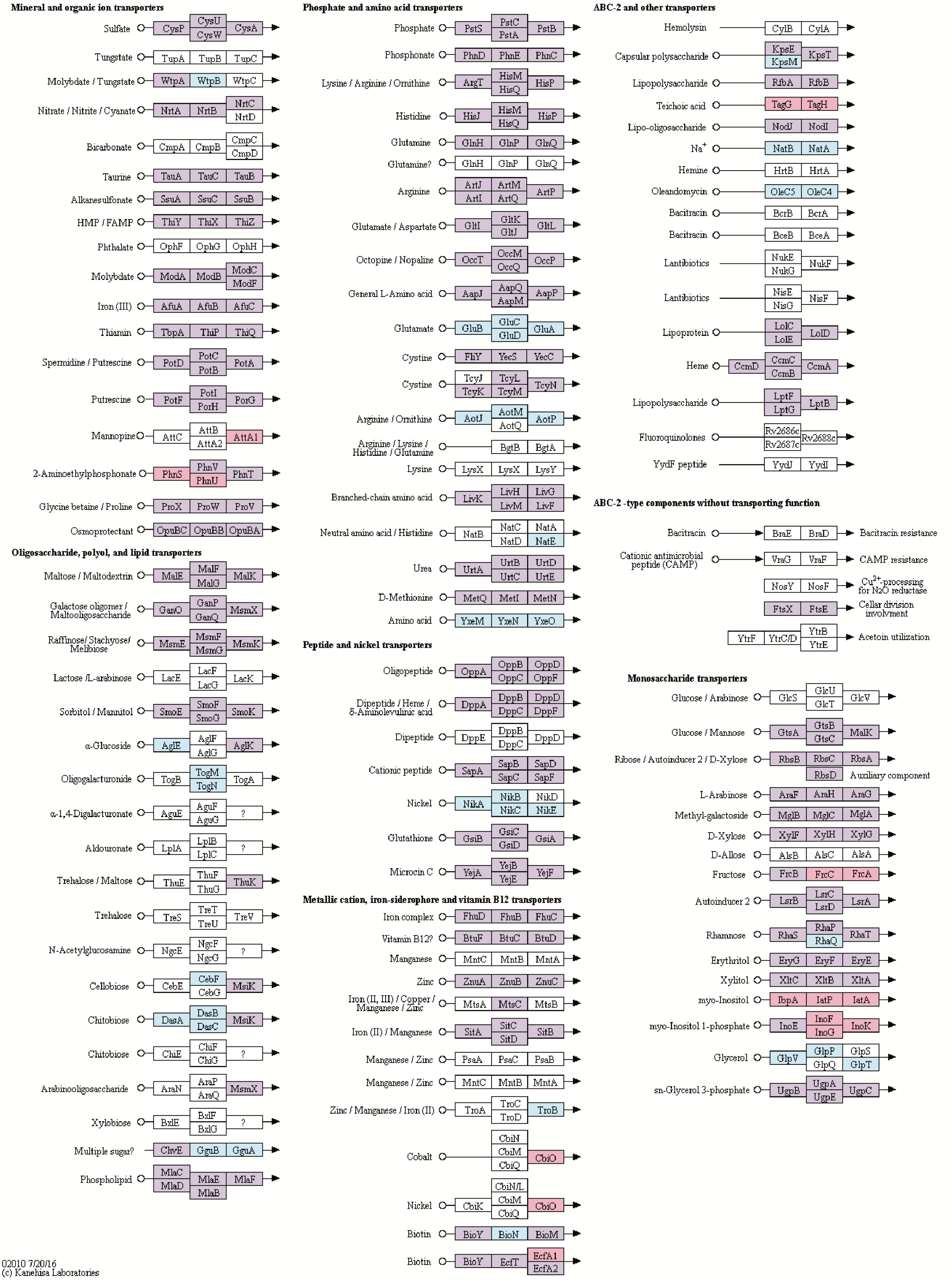

02010 7/20/16

(c) Kanehisa Laboratories
